# Prevalence and predictors of back pain among primary healthcare workers in Qatar in 2023

**DOI:** 10.5339/qmj.2025.100

**Published:** 2025-12-01

**Authors:** Nada Adli Abuhashem, Yara Altahan, Nagah Selim

**Affiliations:** 1 Preventive Medicine Division, Department of Internal Medicine, Hamad Medical Corporation (HMC), Doha, Qatar; 2 Department of Community and Preventive Medicine, Hamad Medical Corporation (HMC), Doha, Qatar; 3 Community and Preventive Medicine Department, Primary Health Care Corporation (PHCC), Doha, Qatar; 4 Public Health and Preventive Medicine, Cairo University, Egypt *Email: nadaadli@outlook.com

**Keywords:** Healthcare workers, PHCC, back pain, predictors, Qatar

## Abstract

**Background:**

Back pain is a common musculoskeletal disorder (MSD) linked to various occupational activities. Understanding its prevalence is essential, as many healthcare workers (HCWs) are at risk of developing back pain and related conditions due to the physical demands of their job responsibilities.

**Methods:**

To determine the prevalence and identify the predictors of back pain and other MSDs among primary HCWs in Qatar in 2023.

**Methods:**

An analytical cross-sectional study was conducted among primary HCWs between May 2023 and June 2023. A multistage random sample (*n* = 654) was used to recruit participants from five healthcare centers nationwide, who were invited to complete a face-to-face survey.

**Results:**

Among the 392 participants, 52% (*n* = 204) reported back pain, with 69% of these cases involving the lower back. Back pain was most common among nurses (21.6%), data entry/computer-based workers (21.1%), office administrative staff (13.2%), and receptionists (11.8%). Other musculoskeletal conditions reported among HCWs included scoliosis (32.6%), right shoulder and knee pain (37.2%), and osteoporosis affecting the neck and spine (9.3%). Logistic regression analysis identified female gender (adjusted odds ratio [AOR]: 2.057; 95% confidence interval [CI]: 1.76–4.85; *p* = 0.0003) and the presence of other MSDs (AOR: 4.695; 95% CI: 2.017–10.929; *p* = 0.000) were significant predictors of back pain.

**Conclusion:**

This study highlights that a substantial proportion of primary HCWs employed in health centers across Qatar experience back pain, representing a serious occupational health concern. Female gender and pre-existing MSDs were identified as key predictors, underscoring the need for targeted interventions. Promoting ergonomic practices and encouraging regular physical activity are essential to reduce back pain and protect the health of HCWs.

## 1. INTRODUCTION

Back pain and musculoskeletal disorders (MSDs) are among the leading causes of disability worldwide, significantly affecting worker productivity and overall well-being.^1^ These conditions also contribute to increased workplace absenteeism, resulting in a substantial economic burden.

Healthcare professionals are at an increased risk for MSDs, including lower back pain (LBP),^1^ with medical staff often exhibiting higher LBP prevalence than other hospital and industrial workers.^1,2^ Nurses, surgeons, and dental professionals experience some of the highest rates of back and musculoskeletal injuries across all occupations. Globally, the prevalence of LBP among healthcare workers (HCWs) is estimated to be as high as 58%.^3^

Nursing is the most extensively studied profession among HCWs regarding back pain and MSDs, primarily due to the physically demanding and labor-intensive nature of the work.^3^ The International Labor Organization (ILO) has classified MSDs as a growing epidemic;^4^ however, research on the subject remains limited, particularly in the Middle East. In 2015, a study conducted in Qatar reported a 54.3% prevalence of LBP among nurses,^5^ while a study from Saudi Arabia found LBP prevalence among HCWs ranging from 48% to 85%.^6^ In India, 92% of physiotherapists reported work-related MSDs,^7^ and similar trends were observed among Italian nurses, with a prevalence reaching 90.2%.^8^

Back pain and MSDs are highly prevalent among HCWs; however, the specific risk factors and individual characteristics that increase an individual’s susceptibility remain unclear.^9^ The literature presents a complex and multifaceted picture, with findings that are often inconsistent.^9^ For example, a study in Italy found that musculoskeletal issues were more common among female nurses but were not associated with years of service, age, or body mass index (BMI).^8^ Additionally, a systematic review identified stress and lack of physical activity as the most significant risk factors for developing MSDs.^9^

Given the varied nature of healthcare work and its interaction with socio-demographic and lifestyle factors, a comprehensive approach to further research is essential to better understand these relationships within specific populations. This study aims to assess the prevalence of back pain symptoms and other MSDs among primary HCWs in Qatar in 2023, as well as the associated risk factors. The findings will contribute to a deeper understanding of the issue and support the development of ergonomic interventions to mitigate its impact.

## 2. METHODS

### 2.1. Study design and setting

An analytical cross-sectional study was conducted in Qatar in 2023 among primary HCWs. The study setting was the Primary Health Care Corporation (PHCC), a governmental institution and the leading provider of primary healthcare services in the country. PHCC comprises its headquarters (administrative offices) and 31 primary healthcare centers distributed across Qatar’s three administrative health regions: North, West, and Central, serving a registered population of more than 1.6 million people.

### 2.2. Sampling

According to the Primary Health Care Corporation Annual Statistical Report for 2020, the total number of HCWs was 6,520.^10^ Assuming a 50% prevalence of back pain, a 95% confidence interval (CI) with a 5% margin of error, and a design effect of 1.0, the minimal required sample size was calculated to be 363 participants using OpenEpi^®^ software version 3.01 based on the following formula:

[Sample size *n* = [DEFF*Np (1−*p*)]/[(*d*^2^/*Z*^2^_1−α/2_*(*N*−1) + p*(1−*p*)].^11^

After adjustment for an anticipated 80% response rate to the face-to-face interviews, the total sample size was determined to be 654 participants.

A multistage sampling technique was initially employed, followed by a simple random sampling method to select participants. The study was conducted across three primary healthcare (PHC) centers, with one center randomly selected from each of the three regions (North, West, and Central), to ensure representation of both clinical and non-clinical staff. In addition, the PHCC administration headquarters, which includes a small health center providing medical services to employees, was included to capture a broader range of job categories, such as accountants, health informatics officers, and clinical coders.

All primary HCWs employed at PHCC for at least one year and able to communicate in either Arabic or English were eligible to participate in the study, with no restrictions on nationality or gender. Participants who provided informed consent after being briefed on the study objectives were included and took part in face-to-face interviews, each lasting approximately 10–15 minutes.

HCWs employed in private centers and hospitals were excluded from this study. Additionally, individuals who had experienced back pain for less than one year, or whose back pain resulted from previous trauma, injury, or accidents unrelated to occupational factors, were not included.

### 2.3. Questionnaire development and validation process

The questionnaire was developed by the author following an extensive literature review. It comprised 14 questions covering socio-demographic and health-related factors, including age, gender, nationality, specialty, years of employment, and daily contact time with patients, materials, or computers. Additional information collected included comorbidities, height, weight, exercise habits, smoking status, and details regarding back pain, its location, and other musculoskeletal issues.

The questionnaire’s face validity was established through consultations with faculty members in preventive medicine and subject-matter experts in the field. Translation validity was achieved by having two native speakers translate the English version into Arabic and then back into English to ensure consistency. All authors reviewed and approved the final version. The questionnaire was then piloted with a sample of 10 HCWs to assess its clarity, comprehensibility, and appropriateness.

### 2.4. Data collection method and variables

**2.4.1. Dependent variables**

The primary outcome variable in this study was the prevalence of back pain, initially assessed as a binary response (Yes or No). Participants who reported back pain were further classified into four specific categories based on the location of the pain: upper back pain, LBP, both upper and lower back pain, and pain in an unspecified location.

**2.4.2. Independent variables**

The data collection tool was used to obtain detailed information on participants’ socio-demographic and background characteristics, including age, gender, nationality, marital status, specialty, professional experience, and daily contact time with patients, materials, or computers.

Additionally, comorbidities were assessed and classified as either present or absent (Yes vs. No). For participants reporting comorbidities, specific conditions such as hypertension, diabetes mellitus, asthma, and hypothyroidism were documented. Participants were also asked about other musculoskeletal problems, which were recorded as a binary response (Yes vs. No).

Furthermore, height and weight measurements were self-reported to calculate BMI, which was then categorized into two groups: normal weight and overweight/obese.

Moreover, information about smoking status was obtained and recorded as either smoker or non-smoker. At the same time, exercise frequency was classified into three categories: 0 hours per week, 1 hour per week, and ≥2 hours per week.

### 2.5. Ethical statement

This study forms part of a larger, yet unpublished project and received approval from the Primary Health Care Corporation Ethical Committee (PHCCIRB) under protocol number PHCC/DCR/2022/10/059. Informed consent was obtained from all participants prior to the interviews. The study was conducted in full accordance with the principles of the “Declaration of Helsinki” and Good Clinical Practice guidelines.

## 3. DATA ANALYSIS

The database was constructed and analyzed using the Statistical Package for the Social Sciences (SPSS)™ Version 25. Descriptive analyses were performed to summarize participants’ characteristics, with categorical variables presented as frequencies and percentages. Additionally, the normality of the dataset distribution was assessed using the Kolmogorov–Smirnov test. The use of either the mean ± standard deviation (SD) or the median ± interquartile range (IQR) depended on the *p*-value obtained from the test.

A chi-squared test was used to examine the significant associations between the socio-demographic and health-related characteristics and carpal tunnel syndrome. Logistic regression was also conducted to identify the independent predictors of carpal tunnel syndrome and to calculate the adjusted odds ratios (AORs) with 95% CI. Two-tailed *p*-values <0.05 were considered statistically significant.

## 4. RESULTS

### 4.1. Sample characteristics

Between May 2023 and June 2023, we approached 654 of the HCWs. A total of 392 participants completed the survey, yielding a response rate of approximately 60%.

### 4.2. Socio-demographic and health-related characteristics of the study participants

The mean age of the HCWs was 37.8 years (SD ± 8.1), and more than half of them (59.2%; *n* = 232) were females. Most of the participants were non-Qatari (89.3%; *n* = 350) and married (77.3%; *n* = 303). Approximately 47% (*n* = 185) of the study sample were clinicians. In addition, 87.5% (*n* = 249) had been working for between 6 and 20 years, and the majority (83.2%; *n* = 326) reported contact with patients, materials, or computers for up to 8 hours/day, as shown in Table 1.

### 4.3. Prevalence of back pain symptoms and their related characteristics among the PHCC HCWs

**Figure 1:**
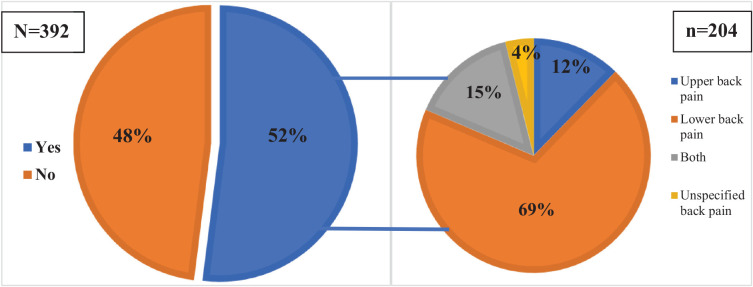
Prevalence and type or site of back pain among PHCC HCWs in Qatar during 2023 (*N* = 392).

Figure 1 illustrates the overall prevalence of back pain among the PHCC HCWs in Qatar during 2023. Notably, 52% of the HCWs reported experiencing back pain. Among those affected, the majority (69%) had lower back pain, 12% had upper back pain, 15% experienced both upper and lower back pain, and 4% reported unspecified back pain.

**Figure 2:**
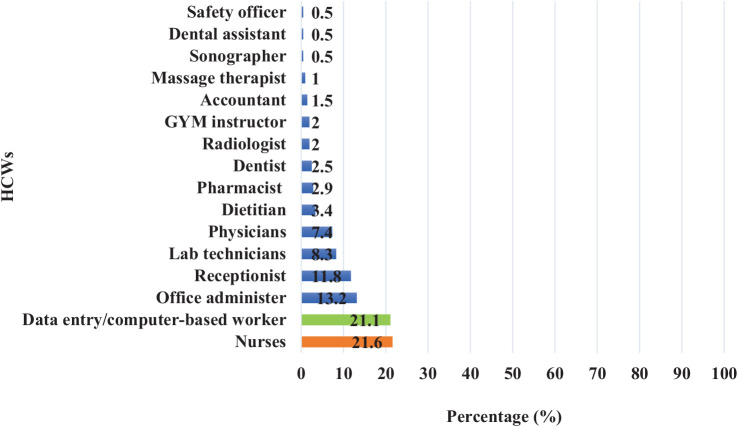
Frequency distribution of back pain according to the nature of work or occupation among PHCC HCWs in Qatar during 2023 (*n* = 204).

Among HCWs reporting back pain, nurses were the most affected, representing 21.6% of cases, followed by data entry and computer-based workers (21.1%), office administrative staff (13.2%), and receptionists (11.8%), as shown in Figure 2.

**Figure 3:**
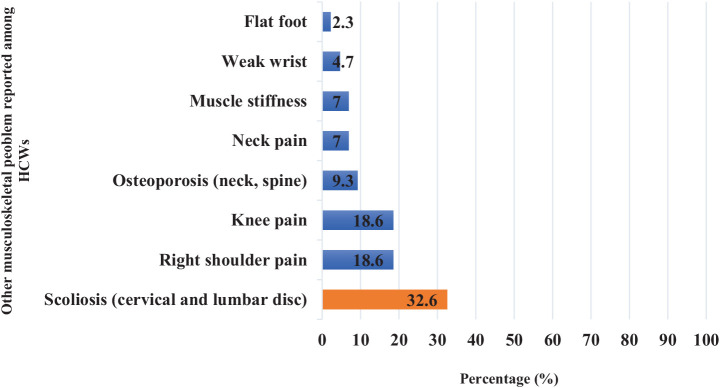
Frequency distribution of other MSDs reported among PHCC HCWs in Qatar in 2023 (*N* = 392).

Other musculoskeletal issues were reported by 43 HCWs (11%), with 32.6% experiencing scoliosis (cervical and lumbar). Additionally, 18.6% reported right shoulder pain, 18.6% had knee pain, and 9.3% reported osteoporosis affecting the neck and spine, as shown in Figure 3.

**Figure 4:**
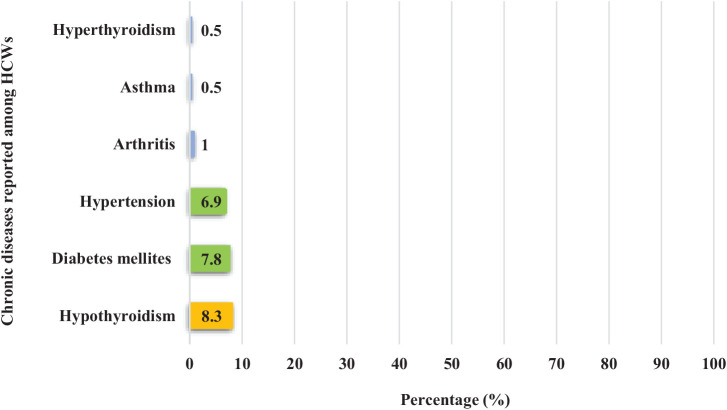
Commonly reported medical illnesses among HCWs with back pain at PHCC in Qatar during 2023 (*n* = 204).

Among the 204 HCWs with back pain, hypothyroidism was the most commonly reported chronic illness, affecting 17 individuals (8.3%), followed by diabetes mellitus (*n* = 16; 7.8%) and hypertension (*n* = 15; 6.9%), as shown in Figure 4.

### 4.4. Potential determinants associated with back pain symptoms

**4.4.1. Socio-demographic and background characteristics associated with back pain symptoms**

Table 2 shows several statistically significant risk factors associated with back pain, including gender, history of chronic disease, and physical activity.

Among the back pain group, female exhibited a higher prevalence (70.6%; *n* = 144) compared to males (29.4%; *n* = 60), with a *p*-value of 0.001.

Additionally, the history of chronic diseases was significantly more prevalent among HCWs with back pain (23.5%) compared to those without back pain (15.4%), with a *p*-value of 0.044.

Furthermore, a higher proportion of HCWs with back pain (27%) reported no physical activity compared to those without back pain (16.5%). Conversely, the percentage of HCWs engaging in more than two hours of physical activity per week was lower among those with back pain symptoms (39.2%) than those without back pain (54.8%). This difference was statistically significant, with a *p*-value of 0.005.

However, no statistically significant differences were observed between the two groups with respect to age, marital status, occupation, years of service, daily contact time with patients/materials/computers, and BMI, as shown in Table 2.

As shown in Table 3, logistic regression analysis revealed that gender, physical activity, and a history of other musculoskeletal problems were significant risk factors for back pain.

Females were found to be nearly three times more likely to develop back pain compared to males, with an AOR of 2.057 (95% CI: 1.76–4.85; *p* = 0.0003).

Additionally, HCWs with other musculoskeletal issues had significantly higher odds of experiencing back pain compared to those without such problems, with an AOR of 4.695 (95% CI: 2.017–10.929; *p* = 0.000), as shown in Table 3.

## 5. DISCUSSION

This study evaluated the prevalence and associated risk factors for back pain among primary HCWs in Qatar in 2023. Back pain is a well-recognized occupational health concern in the healthcare sector, with multiple studies reporting high prevalence rates across various healthcare professions.^9,12^ A systematic review of low back pain among healthcare providers revealed that LBP is widespread, often affecting a significant portion of the workforce, with some healthcare groups experiencing prevalence rates exceeding 66%.^8^

This study identified a back pain prevalence of 52%, with nurses being the most affected group. Comparable findings were observed in Denizli, where 53% of HCWs experienced back pain, predominantly among medical secretaries.^13^ Similarly, a Nigerian study indicated a prevalence of 56.2%.^14^ In Tunisia, approximately 48% of nurses reported low back pain,^15^ while in Serbia, an overwhelming 93% of nurses were affected.^12^

A systematic review of 22 observational studies in Saudi Arabia revealed that nurses and physiotherapists were more susceptible to low back pain,^16^ likely due to the physical demands of their jobs, such as prolonged standing and repetitive bending. The location of pain also varied by profession: German ophthalmologists most commonly reported neck and upper back pain, whereas nurses, physiotherapists, and radiologists experienced LBP due to the specific nature of their work.^17^

Several risk factors for back pain among HCWs have been documented in the literature, including gender, BMI, duration of service, and lack of regular physical activity.^15,16^

The association between gender and low back pain has yielded inconsistent findings. A recent systematic review of 154 studies from various regions worldwide found no significant relationship between gender and low back pain.^9^ Conversely, our study identified a statistically significant difference in low back pain prevalence between females and males, aligning with findings from global systematic reviews and meta-analyses conducted by Hoy et al. (2012).^18^ These studies on MSDs, including low back pain, consistently reported higher prevalence rates among women across different populations and occupational settings compared to men. Additional studies by Bizzoca et al.,^19^ Calais-Ferreira et al.,^20^ and Al Amer^16^ corroborated these findings, attributing the increased prevalence of low back pain among female workers to factors such as physical workload, ergonomic challenges, job demands, and body mechanics.

Furthermore, numerous studies have consistently identified a high BMI as a risk factor for low back pain.^9,14,21^ However, in our study, high BMI was not significantly associated with low back pain. This lack of significance may be attributed to the high prevalence of obesity in Qatar, which could have influenced the observed outcomes.

In addition, insufficient physical activity was identified as a potential risk factor for back pain in the present study, likely reflecting the predominantly sedentary lifestyle of the study population. This finding aligns with the existing literature, which widely recognizes regular physical activity as a protective factor against the onset and persistence of back pain.^9,15,21^ Research conducted in Tunisia and among workers in Saudi Arabia highlighted that older HCWs and those with lower levels of physical activity were at a higher risk of developing back pain.^15,16^ Evidence supports that physical activity effectively reduces LBP among HCWs. Systematic reviews and meta-analyses, including those by van Tulder et al.,^22^ and Hayden et al.,^23^ have demonstrated that exercise therapy—including strength and flexibility exercises—significantly reduces pain and enhances functional outcomes. Specific studies, such as that by Indrayani et al.,^24^ reported significant reductions in the LBP incidence and disability among HCWs who participated in structured exercise programs. Furthermore, occupational health reviews by Linton^25^ highlighted the advantages of incorporating exercise into workplace wellness programs to effectively manage LBP.

## 6. STUDY STRENGTHS AND LIMITATIONS

A notable strength of this study was its inclusion of all HCWs within primary healthcare settings, encompassing both clinical and administrative staff. However, a key limitation lies in its cross-sectional design, which inherently carries the risk of recall and selection bias. Additionally, the reliance on self-reported data—such as BMI—instead of direct measurements may have introduced bias and reduced the accuracy of weight-related variables.

## 7. CONCLUSION AND RECOMMENDATIONS

### 7.1. Conclusion

Back pain is a common occupational hazard among HCWs, affecting 52% of primary HCWs in randomly selected governmental health centers across Qatar in 2023. Female gender, low levels of physical activity, a history of chronic diseases, and other MSDs had emerged as significant risk factors for back pain.

### 7.2. Recommendations

Enhancing ergonomic practices, promoting regular physical activity, and providing training on proper body mechanics are essential to mitigate back pain among HCWs. Additionally, implementing job rotation, adjusting workloads, and offering supportive interventions such as physiotherapy are crucial measures. Regular health screenings and effective management of chronic conditions are also vital for reducing the prevalence of back pain.

## LIST OF ABBREVIATIONS

**Table:** 

BMI	Body Mass Index
HCWs	Healthcare Workers
ILO	International Labor Organization
LBP	Lower Back Pain
MSD	Musculoskeletal Disorder
PHCC	Primary Health Care Corporation/Centers

## DATA AVAILABILITY STATEMENT

The datasets generated and/or analysed during the current study are available from the corresponding author upon reasonable request.

## AUTHORS’ CONTRIBUTION

NA: Conceptualization, methodology, investigation, formal analysis, data curation, project administration, writing—original draft, and review & editing. YA: Conceptualization, writing—original draft, and review & editing. NS: Methodology, formal analysis, data curation, supervision, review & editing.

## CONFLICT OF INTEREST

The authors declare that the research was conducted without any commercial or financial relationships that could be construed as a potential conflict of interest.

## Figures and Tables

**Table 1.** Frequency distribution of the socio-demographic and background characteristics of primary HCWs at PHCC in Qatar during 2023 (*N* = 392).

Socio-demographic characteristics	Frequency (*N* = 392)	Percentage (%)
**Age (years)**		
18–24	15	(3.8)
25–39	232	(59.2)
40+	145	(37.0)
**Mean age (SD) in years**	37.8 ± 8.1	
**Gender**		
Male	160	(40.8)
Female	232	(59.2)
**Nationality**		
Qatari	42	(10.7)
Non-Qatari	350	(89.3)
**Marital status**		
Unmarried	89	(22.7)
Married	303	(77.3)
**Specialty**		
Non-clinical	207	(31.5)
Clinical	185	(41.6)
**Specialty**		
Physician	39	(9.9)
Dentist	11	(2.8)
Lab technician	28	(7.1)
Nurse	75	(19.1)
Office administrators	46	(11.7)
Receptionist	34	(8.67)
Data entry/computer-based work	112	(28.5)
Accountant	5	(1.27)
Sonographer	1	(0.25)
Radiologist	4	(1.02)
Massage therapy	2	(0.5)
Dietitian	12	(3.06)
Wellness counselor	2	(0.5)
GYM instructor	6	(1.5)
Dentist assistant	1	(0.25)
Pharmacist	10	(2.5)
Safety officer	4	(1.02)
**Occupation (years)**		
0–5	111	(28.3)
6–10	96	(24.5)
11–15	88	(22.4)
16–20	65	(16.6)
21+	32	(8.2)
**Contact time with patient/material/computer**		
Up to 8 hours/day	326	(83.2)
More than 8 hours/day	66	(16.8)

**Table 2.** Association between back pain and demographic and health-related characteristics among the PHCC HCWs in Qatar in 2023 (*n* = 392).

Back pain-related variables	Group Back pain				*p*-value
	Yes (*n* = 204)		No (*n* = 188)		
	*n*	(%)	*n*	(%)	
**Age (years)**					
≤39	123	(60.3)	124	(66.3)	0.366
40+	81	(39.7)	64	(34.0)	
**Mean age (SD) in years**	38.1 ± (8.3)		37.40 ± (7.7)		0.344
**Gender**					
Male	60	(29.4)	100	(53.2)	0.000*
Female	144	(70.6)	88	(46.8)	
**Marital status**					
Unmarried	47	(23.0)	42	(22.3)	0.869
Married	157	(77.0)	146	(77.7)	
**Specialty**					
Non-clinical	102	(50.0)	105	(44.1)	0.246
Clinical	102	(50.0)	83	(55.9)	
Occupation					
0–5 years	54	(26.5)	57	(30.3)	
≥6 years	150	(73.5)	131	(69.7)	0.171
**Contact time with patient/material/computer**					
Up to 8 hours/day	171	(83.8)	155	(82.4)	0.716
More than 8 hours/day	33	(16.2)	33	(17.6)	
**History of chronic Disease**					
Yes	48	(23.5)	29	(15.4)	0.044**
No	156	(76.5)	159	(84.6)	
**Obesity**					
Yes	176	(86.7)	154	(82.8)	0.284
No	27	(13.3)	32	(17.2)	
**Smoking status**					
Smoker	17	(8.3)	15	(8.0)	0.898
Non-smoker	187	(91.7)	173	(92.0)	
**Exercise**					
0 hours/week	55	(27.0)	31	(16.5)	
1 hour/week	69	(33.8)	54	(28.7)	0.005***
≥2 hours/week	80	(39.2)	103	(54.8)	

*p < 0.001.

**p < 0.05.

***p < 0.01.

**Table 3.** Logistic regression between the socio-demographic and background characteristics and back pain symptoms among the PHCC HCWs in Qatar during 2023 (*N* = 392).

Constant	*p*-value	Adjusted odds ratio	95% CI	
			Lower	Upper
**Age (years)**				
≤39 [Reference]				
40+	0.101	0.614	0.343	1.100
**Gender**				
Male [Reference]				
Female	0.000***	2.930	1.769	4.852
**Specialty**				
Clinicians [Reference]				
Non-clinicians	0.438	0.835	0.530	1.316
**Occupation**				
0–5 years [Reference]				
≥6 years	0.303	1.345	0.784	2.310
**Contact time with patient/material/computer**				
Up to 8 hours/day [Reference]				
More than 8 hours/day	0.438	1.266	0.698	2.295
**Obesity**				
No [Reference]				
Yes	0.122	1.670	0.872	3.199
**Smoking**				
No [Reference]				
Yes	0.082	2.057	0.912	4.641
**Exercise**				
Yes [Reference]				
No	0.074	1.625	0.954	2.767
**Other musculoskeletal problems**				
No [Reference]				
Yes	0.000***	4.695	2.017	10.929

*p < 0.05; **p < 0.01; ***p < 0.001.
